# Amelioration of Chlorpyrifos-Induced Toxicity in *Brassica juncea* L. by Combination of 24-Epibrassinolide and Plant-Growth-Promoting Rhizobacteria

**DOI:** 10.3390/biom11060877

**Published:** 2021-06-12

**Authors:** Palak Bakshi, Rekha Chouhan, Pooja Sharma, Bilal Ahmad Mir, Sumit G. Gandhi, Marco Landi, Bingsong Zheng, Anket Sharma, Renu Bhardwaj

**Affiliations:** 1 Department of Botanical and Environmental Sciences, Guru Nanak Dev University, Amritsar 143005, India; palakbot.rsh@gndu.ac.in (P.B.); poojas3184@gmail.com (P.S.); 2 Indian Institute of Integrative Medicine (CSIR-IIIM), Council of Scientific and Industrial Research, Jammu 180001, India; imrekha42@gmail.com (R.C.); sumit@iiim.res.in (S.G.G.); 3 Department of Botany, School of Life Science, Satellite Campus, University of Kashmir, Kargil, Jammu and Kashmir 190006, India; meerbilal82@gmail.com; 4 Department of Botany, Kargil Campus, Khumbathang-Kargil, University of Ladakh, Ladakh 194105, India; 5 Department of Agriculture, Food and Environment, University of Pisa, I-56124 Pisa, Italy; marco.landi@unipi.it; 6 CIRSEC—Centre for Climatic Change Impact, University of Pisa, I-56124 Pisa, Italy; 7 State Key Laboratory of Subtropical Silviculture, Zhejiang A&F University, Hangzhou 311300, China; bszheng@zafu.edu.cn

**Keywords:** plant-growth-promoting rhizobacteria, chlorpyrifos, 24-epibrassinolide, *Brassica juncea*, reactive oxygen species, nitrate reductase, nitric oxide

## Abstract

Pervasive use of chlorpyrifos (CP), an organophosphorus pesticide, has been proven to be fatal for plant growth, especially at higher concentrations. CP poisoning leads to growth inhibition, chlorosis, browning of roots and lipid and protein degradation, along with membrane dysfunction and nuclear damage. Plants form a linking bridge between the underground and above-ground communities to escape from the unfavourable conditions. Association with beneficial rhizobacteria promotes the growth and development of the plants. Plant hormones are crucial regulators of basically every aspect of plant development. The growing significance of plant hormones in mediating plant–microbe interactions in stress recovery in plants has been extensively highlighted. Hence, the goal of the current study was to investigate the effect of 24-epibrassinolide (EBL) and PGPRs (*Pseudomonas aeruginosa* (Ma), *Burkholderia gladioli* (Mb)) on growth and the antioxidative defence system of CP-stressed *Brassica juncea* L. seedlings. CP toxicity reduced the germination potential, hypocotyl and radicle development and vigour index, which was maximally recuperated after priming with EBL and Mb. CP-exposed seedlings showed higher levels of superoxide anion (O_2_^−^), hydrogen peroxide (H_2_O_2_), lipid peroxidation and electrolyte leakage (EL) and a lower level of nitric oxide (NO). In-vivo visualisation of CP-stressed seedlings using a light and fluorescent microscope also revealed the increase in O_2_^−^, H_2_O_2_ and lipid peroxidation, and decreased NO levels. The combination of EBL and PGPRs reduced the reactive oxygen species (ROS) and malondialdehyde (MDA) contents and improved the NO level. In CP-stressed seedlings, increased gene expression of defence enzymes such as superoxide dismutase (SOD), ascorbate peroxidase (APOX), glutathione peroxidase (GPOX), dehydroascorbate reductase (DHAR) and glutathione reductase (GPOX) was seen, with the exception of catalase (CAT) on supplementation with EBL and PGPRs. The activity of nitrate reductase (NR) was likewise shown to increase after treatment with EBL and PGPRs. The results obtained from the present study substantiate sufficient evidence regarding the positive association of EBL and PGPRs in amelioration of CP-induced oxidative stress in *Brassica juncea* seedlings by strengthening the antioxidative defence machinery.

## 1. Introduction

The rising demand for food for the exponentially growing population of the world, which is projected to reach the 9.8 billion mark by 2050 [1], forces the world’s agricultural systems to adapt different channels to enhance the yield [2]. Around 3.13% of the world’s total land is suitable for agriculture, and only 38.47% of that is used primarily for farming [3]. Pesticides are being used at an unprecedented rate around the world to meet the demand and to ensure the quality of vegetation. The intensive, repeated, persistent and desultory intake of diverse pesticides, on the other hand, pollutes the environment with their leftovers [4]. It has been anticipated that less than 0.3% of the pesticides administered target the intended species, while the other 99.7% of the pesticides get accumulated. Pesticidal residues present in the environment eventually find a way to enter the food chain, exposing the whole ecosystem [5].

Since the 1960s, organophosphates (OPs) have floated in the market to combat pest attacks and are now a widely used pesticide due to their moderate toxicity [6]. It has been estimated that OPs share over 36% of the total global pesticide industry [7]. CP, an organophosphorus pesticide, has ranked first amongst the other conventionally used pesticides [8]. CP has been in use on a large number of economically vital crops such as cereals, vegetables, fruits, tobacco and others to remove a wide range of pests such as leaf folders, leaf hopper, flies, termites, cockroaches, beetles, fire ants and nematodes [9,10]. However, the increasing risk of phytotoxicity caused by pesticidal residue accumulation in non-targeted species as well as in soil, water (ground and surface) and air [11,12,13], leading to various human health issues [14], outweighs the benefits offered by the pesticides. Agrochemical application influences the seed germination potential and impacts a variety of physiological and biochemical processes, such as disrupting membrane integrity, enzyme function and nucleic acid damage [15,16]. ROS is considered as an index to depict cell damage. A recent study conducted by Liu and Zhu [5] demonstrated the significant increase in ROS in *Oryza sativa* L. leaves under pesticidal stress. An enhanced level of MDA in oxidative stress conditions acts as a marker in measuring cellular damage. In *Brassica juncea* L. and *Trigonella* seedlings an elevated level of O_2_^−^, H_2_O_2_ and MDA has been observed under imidacloprid, tricyclazole, thiabendazole and plethora toxicity [17,18]. Prolonged exposure and accumulation of ROS leads to oxidative burst in plants, which results in cellular damage, lipid peroxidation, protein degradation and cell death. Increased lipid peroxidation causes electrolyte leakage, which disrupts the ion equilibrium [19]. Plants are dynamic members of a multicellular complex community that have the ability to adapt themselves continuously through changing environmental conditions. The robust innate defence mechanisms of plants counteract the generated ROS and oxidative stress by stimulating the antioxidative defence enzymes. SOD, peroxidase (POD), CAT, APOX and DHAR play key roles in combating pesticidal toxicity in plants [20,21]. NR-dependent production of NO boosts the antioxidative response of the plant under stressed conditions [22]. In *Brassica* seedlings, it also plays a key role in regaining overall plant growth through the signalling network [23].

*Brassica juncea* L. is a high-fibre, mineral-rich and phytochemical-rich food crop that produces oil [24]. During its complete life cycle, it is targeted by various pests such as aphids, termites, leaf hoppers, etc. [25]. Plant hormones are growth regulators which play a prominent role in providing a plant defence against stressful conditions. Brassinosteroids (BRs), a class of steroidal hormones, significantly contributes to plant growth and developmental processes such as elongation of cells, germination potential of seeds, vascular differentiation, photosynthetic activity, rhizogenesis, reproductive phase, senescence, etc. [26]. BR activates the signalling cascade and alters the expression of different genes which are involved in various physiological functions of plants [27]. The growth-stimulatory role of BRs has been well documented by Nolan et al. [28]. In CP-stressed plants, foliar application of EBL alleviates the toxic effect by enhancing the root and shoot elongation [29]. BRs have the potential to revert pesticide-induced toxicity by protecting the plants through boosting the antioxidative defence machinery [30]. Seed priming with EBL decreases the lipid peroxidation, EL and H_2_O_2_ in *Cucumis sativus* [31]. Application of EBL reduced the accumulation of ROS by altering the expression of antioxidative defence enzymes [29] and production of NO via NR [22]. An investigation by Kaya et al. [32] revealed that the potential role of EBL in stimulating NR activity for the production of NO strengthens the antioxidative defence mechanism in stressed pepper plants.

Plants serve as a functional connection between the above-ground and below-ground populations [33] that interact directly or indirectly. Plants’ root systems interact with the rhizosphere, which is one of the most important mechanisms for their survival. The rhizospheric zone consists of a highly diverse niche which extends the ability of plants to survive by increasing soil minerals access. From previous studies, it has been speculated that plants have sophisticated mechanisms to connect with the root-associated microflora by secreting root exudates [34,35]. Plant-growth-promoting rhizobacteria (PGPRs) play a vital role in plant growth and overall development by shaping the chemistry of the rhizosphere and providing pathogen tolerance, increased nutrient absorption, plant biomass, etc. [36]. Supplementation of PGPRs in pesticide-contaminated soil is the most encouraging approach [37] to remediate the pesticidal contamination. Application of plant-growth-promoting traits containing *Bacillus* strains improves the growth of pepper seedlings grown in the presence of fluopyram [38]. Different genera of bacteria such as *Streptomyces* [39], *Pseudomonas* [40,41,42], *Klebsiella* [43], *Bacillus* [44,45,46] and *Burkholderia* [47] have been identified which have the potential to degrade the pesticide efficiently. A study conducted by Khanna et al. [48] documented the ameliorative effect of PGPRs on the growth of tomato plant by upregulating the defence mechanism of the plants under stressed conditions. A positive growth response of strawberry has been observed when inoculated with *Azospirillum brasilense*, *Burkholderia cepacian* or *Enterobacter cloacae* [49]. In wheat plant, *Pseudomonas aeruginosa* promotes plant growth by reducing oxidative stress, by scavenging ROS through increased antioxidative defence enzymes under stressed conditions [50,51]. PGPRs strengthen the plant defence mechanism and improve the plant health by alleviating the effect of pesticidal stress on plants [52].

Interactions between plants and microbes cause significant changes in root and shoot development, architecture and stress tolerance that are regulated by plant hormones [53]. The promising role of plant hormones has been explored in shaping the plant–microbe interaction in order to support the plant under stressed conditions [54,55]. Plant hormones have a direct or indirect effect on the plant microbiome and their interactions with microbes, which is beneficial to the plant’s survival [56]. Keeping in mind the beneficial role of BRs and PGPRs, this study was designed to understand the effect of 24-epibrassinolide-mediated plant PGPRs (*Pseudomonas aeruginosa*, *Burkholderia gladioli*) response on physiological processes of CP-stressed *Brassica juncea* seedlings using UV-Vis spectrophotometer and their expression at the transcript level using an RT-PCR system.

## 2. Materials and Methods

### 2.1. Treatment with Microbes

*Pseudomonas aeruginosa* (MTCC7195, Ma) and *Burkholderia gladioli* (MTCC10242, Mb) were procured from IMTECH (Punjab, India). These plant-growth-promoting strains were revived from lyophilised vials in 50 mL of HiMedia (Mumbai, India) nutrient broth and incubated in a Caltan Deluxe Automatic (New Delhi, India) BOD incubator at 28 °C for 48 h. For future use, the cultured growth was sub-cultured in a timely manner. For the current study, culture was centrifuged (8000 rpm, 4 °C, 15 min) for the formation of pellet, which was washed twice and resuspended in double-distilled water (DDW) to obtain 10^9^ cells/mL.

### 2.2. Plant Material and Experimental Plot

Certified seeds of *Brassica juncea* L. (Var. RLC-3) were acquired from Punjab Agriculture University, Ludhiana. Seeds were first surface sterilised (0.5%*v*/*v* sodium hypochlorite) followed by repeated 2–3 times washing with DDW. From preliminary testing, the effective concentration of EBL (100 nM) that resulted in the most plant growth was chosen. Seeds were pre-sown in 100 nM EBL for 8 h in the dark. With-EBL (EBL)-treated, and without-EBL (WEBL)-treated seeds were placed on autoclaved petri-plates lined with Whatman #1 with CP (IC50- 500 mg/L) and supplemented with Ma or Mb, which were selected based on the results of earlier laboratory work by Khanna et al. [48]. We observed the petri-plates for 10 days in a seed germinator which was maintained at 25 ± 2 °C with a light intensity of 175 μmol m^−r^ s^−s^ and a photoperiod of 16 h before executing the experiment in field conditions. The seedlings were harvested, washed thoroughly with distilled water and processed for analysis.

All of the experiments for each treatment were conducted on biological replicates in triplicate, with one petri representing one repeat and three technical copies for each biological replicate. All the analysis for each treatment was presided over on biological replicates in triplicate by considering one petri as one replicate, and for each biological replicate we took three technical replicates.

### 2.3. Analysis of Growth Parameters

The analysis of growth parameters was done in terms of germination potential, hypocotyl and radicle length and overall growth in terms of vigour index in 10-day-old *B. juncea* seedlings.

Germination potential (%) and vigour index were calculated with the formula described in [57,58]:

$$Germination\;potential\;\left(\%\right)=\frac{\mathrm{Number}\;\mathrm{of}\;\mathrm{seeds}\;\mathrm{germinate}\;\times 100}{\mathrm{Total}\;\mathrm{number}\;\mathrm{of}\;\mathrm{seeds}}$$

Vigour Index = [(Hypocotyl length + Radicle length) × Germination potential (%)]

### 2.4. Cell Injury

Cell injury was determined by measuring the membrane permeability via electrolyte leakage, which was measured using the conductivity meter according to the method given by Lu et al. [59]. Seedlings were incubated overnight at 10 °C by positioning them in 20 mL double-distilled water test tubes. Electrical conductivity (EC-1) of the samples was measured and EC-2 was determined by first autoclaving the samples at 120 °C for 15 min. EL percentage (EL %) was calculated by following the given formula.

Electrolyte Leakage (%) = (EC1/EC2) ×100

### 2.5. Analysis of Oxidative Stress Markers

#### 2.5.1. Superoxide Anion (O_2_^−^)

The O_2_^−^ content was estimated spectrophotometrically by following the method given by Wu et al. [60]. Briefly, one gram of fresh plant sample was grounded in phosphate buffer (6 mL of 65 mM, pH 7.8) containing 2% polyvinylpyrrolidone, followed by centrifugation (at 5000× *g* for 15 min at 4 °C). The mixture was incubated at 25 °C for 30 min. After that, 0.5 mL of supernatant was mixed with 0.5 mL of phosphate buffer and 0.1 mL of hydroxylamine hydrochloride (10 mM, Sigma Alrdrich, Mumbai, India) and incubated again. Incubated mixture was than mixed with 3-amino benzene sulphonic acid and 1-naphthylamine (1 mL of 58 and 7 mM respectively, CDH, New Delhi) and kept at 25 °C for 20 min. The absorbance was recorded at 530 nm and the amount of O_2_^−^ was calculated against the sodium nitrite (Himedia, Mumbai, India) as standard which was expressed as μmol g^−1^ FW. The in-vivo visualisation of O_2_^−^ was done by using the method mentioned by Frahry and Schopfer [61]. The leaves of *B. juncea* were first incubated in nitro blue tetrazolium (NBT, Himedia, Mumbai, India) prepared in potassium phosphate buffer with pH 6.4 and sodium azide (10 mM), followed by a process of decolouration (by immersing them in boiling ethanol), and photographed.

#### 2.5.2. Hydrogen Peroxide (H_2_O_2_)

The H_2_O_2_ content was analysed as per the method proposed by Patterson et al. [62]. 0.5 g of fresh sample was crushed in acetone (1 mL) and centrifuged (at 5000× *g* for 20 min at 4 °C). 20 µL of 20% titanium chloride in conc. HCl was added to the supernatant, followed by addition of ammonium sulphate (200 µL of 17 M, (Himedia, Mumbai, India). The precipitates formed were washed thoroughly with acetone and finally dissolved in 1.5 mL of 2 N H_2_SO_4_ (Himedia, Mumbai, India). The absorbance of the sample was measured at 410 nm. The content of H_2_O_2_ was computed by taking H_2_O_2_ as standard and recorded as μmol g^−1^ FW. Visualisation of accumulation of H_2_O_2_ content in leaves was done by following the protocol stated by Thordal and Christensen [63] in which leaves were immersed in 3,3′-diaminobenzidine (1%, DAB, Sigma Alrdrich, Mumbai, India) in the dark, followed by decolouration to clearly visualise the brown spots. H_2_O_2_ content in *B. juncea* roots was visualised by staining them with 2′,7′-dichlorofluorescin diacetate (DCF-DA, Sigma Alrdrich, Mumbai, India), following the method given by Rodriguez-Serrano et al. [64].

### 2.6. Estimation of MDA Content

MDA content was assayed by employing the method of Heath and Packer [65]. One gram of seedlings was homogenised in trichloroacetate (0.1%, TCA, Himedia, Mumbai, India ), followed by centrifugation (at 13,000× *g* for 15 min at 4 °C). Supernatant was than mixed with a mixture of TCA (20% TCA and 0.5% thiobarbituric acid, Himedia, Mumbai, India) and incubated for 30 min at 95 °C, followed by instant cooling of the mixture in an ice bath. Absorbance of the supernatant was read at 600 and 532 nm. The content was calculated by subtracting the absorbance (600–532 nm) and using 155 mM^−1^ cm^−1^ as the extinction coefficient. In vivo visualisation of lipid peroxidation was performed by following the method of Pompella et al. [66]. Evans’s blue stain was used to visualise the membrane integrity by following the method given by Yamamoto et al. [67]. Cotyledons and root tips of the seedlings were dipped in Schiff’s reagent (Himedia, Mumbai, India) and 0.025% *w*/*v* Evans’s blue (Himedia, Mumbai, India) prepared in 100 μM CaCl_2_ of pH 5.6 for 20 min, followed by bleaching via putting them in boiling ethanol, and photographed.

### 2.7. Fluorescent Imaging of Nuclear and Membrane Damage

The nuclear and membrane damage was examined according to the procedure of Gutierrez-Alcala et al. [68] and Callard et al. [69]. The roots of *B. juncea* seedlings were dipped in propidium iodide (50 μM, Sigma-Aldrich, Mumbai, India). Afterwards, they were washed with double-distilled water and finally mounted on water for examination under fluorescent microscopy (excitation—543 nm, emission—617 nm). For visualisation of membrane damage, the roots were treated with 4,6-diamino-2-phenylindole (0.1 mg in 100 mL PBS) and incubated in the dark for 30 min followed by washing with PBS, and water-mounted slides were used for further examination (excitation—358 nm, emission—461 nm) under a fluorescent microscope (Nikon eclipse Ti-2, Japan).

### 2.8. Estimation of Nitrate Reductase and NO Content

Nitrate reductase activity (NR. E.C. 1.7.1.1) was estimated using the Jaworski [70] method. *B. juncea* seedlings were crushed in phosphate buffer (100 mM, pH 7.5), KNO_3_ (200 mM) and isopropanol (5%), followed by incubation for 2 h. Sulphanilamide (1%) and N-(1-Naphthyl) ethylene diaminedihydrochloride (NED-HCl, 0.02%) were prepared and the enzyme extract was added to initiate the reaction and further incubated at room temperature for 20 min. The optical density was recorded at 540 nm. NaNO_2_ was used as standard to calculate the activity of NR. 

NO content was measured by using the procedure given by Zhou et al. [71]. Seedlings were homogenised in Zinc acetate (4%) containing acetic acid buffer (50 mM, pH 3.6), followed by centrifugation at 4 °C for 15 min at 10,000× *g*. The supernatant was added to the reaction mixture containing Griess reagent (Sigma Alrdrich, Mumbai, India) and charcoal. The optical density was recorded at 540 nm and NaNO_2_ was utilised as standard to calculate NO content. Visualisation of NO in *B. juncea* roots was also carried out by following Rodriguez-Serrano et al. [64]. Roots of *B. juncea* were dipped for 1 h in 5,6-diaminofluorescein diacetate (10 μM, DAF-2 DA, Sigma-Aldrich, Mumbai, India) and were washed thoroughly with distilled water and finally water mounted on glass slides. The imaging of fluorescence was recorded with a fluorescent microscope (Nikon eclipse Ti-2, Tokyo, Japan).

### 2.9. Antioxidative Defence System

For estimation of enzyme activities, the plant elixir was prepared by homogenising the fresh seedlings (1 g) in pre-cooled potassium phosphate buffer (3 mL) with different concentrations and pH.

For estimation of catalase (CAT, EC 1.11.1.6), the standard protocol of Aebi [72] was referred to, to measure the decomposition rate of H_2_O_2_. Plant sample was grounded in 50 mM phosphate buffer with neutral pH. The decrease in absorbance at 240 nm was observed by adding enzyme extract to 15 mM H_2_O_2_ in buffer (PPB, 50 mM, pH-7.0). The ε = 39.4 mM^−1^ cm^−1^ was used as the extinction coefficient. 

Ascorbate peroxidase (APOX, EC 1.11.1.11) activity was assayed by following the protocol of Nakano and Asada [73]. An enzyme extract was prepared by grinding the plant sample in 50 mM, pH 7.0 phosphate buffer. The reaction mixture was prepared by mixing 1 mM H_2_O_2_ and 0.5 mM ascorbate in buffer (PPB, 50 mM, pH 7.0). Enzyme extract was added to the reaction mixture and decrease in absorbance at 290 nm per minute was measured. The extinction coefficient used was ε = 2.8 mM^−1^ cm^−1^.

The activity of glutathione peroxidase (GPOX, EC 1.11.1.9) was analysed using the Flohe and Gunzler [74] method. Plant extract prepared in 50 mM, pH 7.0 phosphate buffer was added to the reaction mixture comprising PPB (50 mM, pH 7.0), GSH (1 mM), sodium azide (1 mM), H_2_O_2_ (0.15 mM), NADPH (0.15 mM), EDTA (0.5 mM) and GR (2.4 units/mL). The change in optical density was measured at 340 nm (ε = 6.22 mM^−1^ cm^−1^).

Glutathione reductase (GR, EC 1.8.1.7) activity was based on methodology given by Carlberg and Mannervik [75]. Plant elixir prepared in 50 mM of buffer with pH 7.8 was used as enzyme extract and added to the reaction mixture with PPB (50 mM, pH 7.8), 1 mM EDTA, NADPH (0.1 mM) and glutathione peroxidase (1 mM, Himedia, Mumbai, India). The optical density was recorded at 340 nm and the extinction coefficient ε = 6.22 mM^−1^ cm^−1^ was used. 

Dehydroacorbate reductase (DHAR, EC 1.8.5.1) activity was analysed by the method given by Dalton et al. [76]. Homogenisation of plant sample was done in phosphate buffer (100 mM, pH 7.0). The reaction buffer contained glutathione reductase (2.5 mM, Himedia, Mumbai, India), PPB (50 mM, pH 7.0), dehydroascorbate (0.2 mM), EDTA (0.1 mM) and plant sample. The absorbance was recorded at 265 nM and ε = 14 mM^−1^ cm^−1^ was utilised to determine the content.

The superoxide dismutase (SOD, EC1.15.1.1) content was measured using Kono’s [77] protocol. The sum of enzyme vital for 50% inhibition of reduction in NBT per unit per g of fresh weight is referred as one unit of SOD activity. The enzyme extract for determination of SOD was prepared in 50 mM Na_2_CO_3_ buffer with pH 7.8. Enzyme extract was added after 2 min of initiation of reaction. The reaction was initiated when 1 mM of hydroxylamine hydrochloride was added to the reaction mixture consisting of 0.03% of triton X-100, 24 μM nitro blue tetrazolium, EDTA (0.1 mM) and Na_2_CO_3_ buffer (50 mM, pH 7.8). The decrease in absorbance at 560 nM for 2 min was recorded due to inhibition of reduction in NBT.

### 2.10. Analysis of Gene Expression through qRT-PCR

RNA isolation from the seedlings was done using the TRIzol method (Invitrogen, Life Technologies, Waltham, MA, USA) by following the instructions given by the manufacturer. A nanodrop spectrophotometer (Thermo Scientific, Waltham, MA, USA) was used to quantify the isolated RNA followed by a quality check on 2% agarose gel electrophoresis. RNA to cDNA was synthesised by following Awasthi et al. [78]. qRT-PCR quantification was done using ROTOR geneq RT-PCR system. Reaction mixture contained SYBR green, gene-specific primer (designed with Primer 3 software, Table 1) and cDNA. Actin was used as a housekeeping gene and each assay was performed in triplicate. Ct value was used for calculating the relative expression of a gene by using the 2^−ΔΔct^ method [79].

### 2.11. Statistical Analysis

The experiments were performed in triplicate and the results presented are in means ± S.D. in figures. Statistical analysis was performed using two-way analysis of variance (ANOVA) on biochemical parameters and one-way ANOVA on gene expression analysis using SPSS 16.0. Tukey’s alpha test was used for the separation of means among the treatments (*p* < 0.05 level of significance). Pearson correlation analysis was performed to find the associations among the different morphological and biological parameters using R software v3.0 (Statistical Computing, Vienna, Austria). Heatmap was also performed using R software to check the variations of gene expression with respect to different treatments.

## 3. Results

### 3.1. Effect of EBL and PGPRs on Growth of Brassica juncea Seedlings Treated with CP

**Figure 1:**
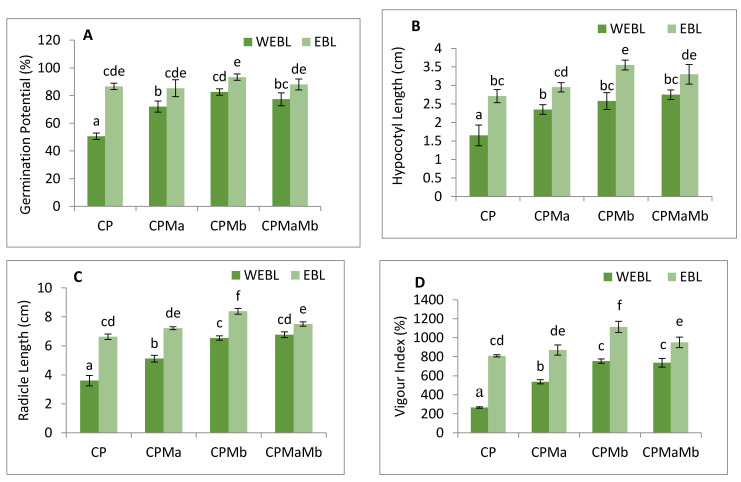

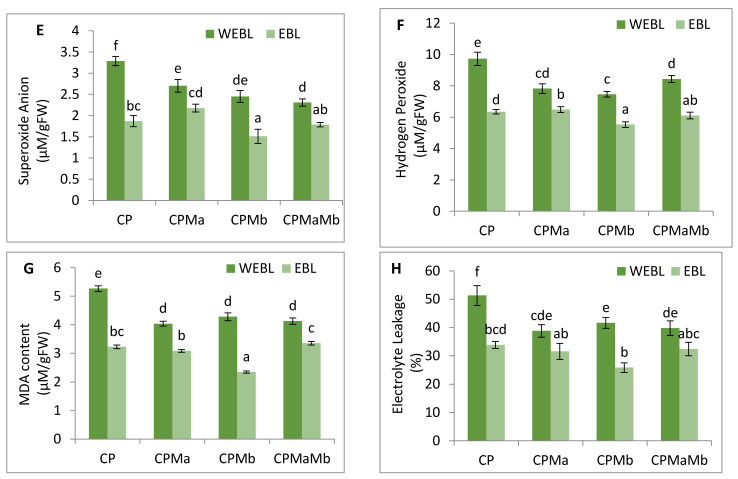
Effect of seed priming with 100 nM of 24-epibrassinolide (EBL) and plant-growth-promoting rhizobacteria (*Pseudomonas aeruginosa* (Ma, 10^9^ cells/mL) and *Burkholderia gladioli* (Mb, 10^9^ cells/mL)) on (**A**) germination potential (%), (**B**) hypocotyl length (**C**) radicle length, (**D**) vigour index (%), (**E**) superoxide anion, (**F**) hydrogen peroxide, (**G**) MDA content and (**H**) EL content of 10-day-old *B. juncea* L. seedlings under chlorpyrifos (CP) toxicity. Data are presented as mean of three replicates ± s.d. (standard deviation). Means were compared using two-way analysis of variance followed by Tukey’s post-hoc test (*p* < 0.05). Different superscripts indicate significant difference between the treatments. (Note: WEBL = *without 24-epibrassinolide*, EBL = *24-epibrassinolide*).

The annotations prepared on the growth profile of *B. juncea* to assess the effect of EBL and PGPRs under CP stress were measured in requisites of germination potential (Figure 1A), hypocotyl growth (Figure 1B) and radicle growth (Figure 1C). The vigour index was used to track the overall growth of the seedlings (Figure 1D). Decreased germination potential, seedling growth and vigour index were observed when CP-treated seedlings were compared in contrast to without CP. However, seed pre-soaking with EBL along with supplementation with Ma and Mb alone and in combinations, i.e., EBLMaMb significantly recovered the seedlings’ growth. Treatment of EBL (100 mM) enhanced the germination potential (41.57%), hypocotyl length and radicle length (39.1 and 45.6%) when compared with CP only. However, supplementation of Ma along with CP, Mb with CP and together in a combination of Ma and Mb along with CP also improved the growth profile of the seedlings when compared with CP only. The sharpest rise was seen in the germination potential by 45.7%, hypocotyl length by 53.5%, radicle by 57% and overall growth, i.e., vigour index by 76.1% when Mb was inoculated with EBL treatment and compared with CP-treated seedlings. The data was statistically analysed by using two-way ANOVA which showed a significant difference in the various growth attributes. The results show that CP stress considerably impeded seedling growth, which was positively influenced by supplementation with EBL and PGPRs, showing that EBL and PGPRs had an ameliorative impact.

### 3.2. Effect of EBL and PGPRs on Oxidative Damage in Brassica juncea Seedlings Treated with CP

**Figure 2:**
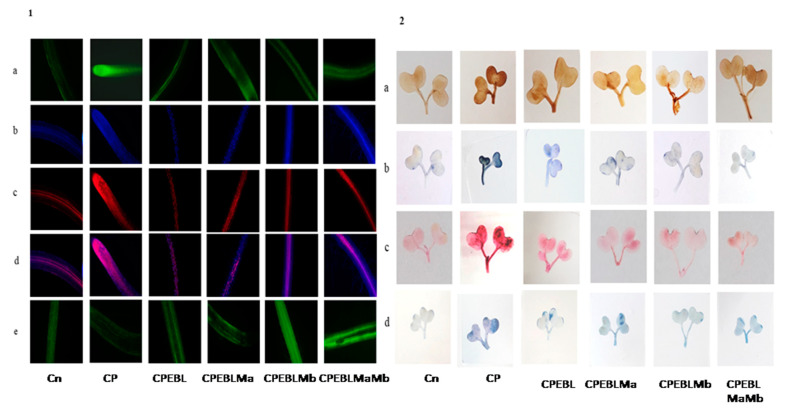
(**1**) In situ visualisation of *B. juncea* roots indicating the generation of: (**a**) H_2_O_2_ tagged with DCF-DA, the green color intensity represents the level of H_2_O_2_; (**b**) nuclear damage exposed with DAPI, the blue dots represent the nuclear damage; (**c**) membrane damage tagged with PI, the red color dots represents the membrane damage; (**d**) their interlay, dark pink color represent the maximum nuclear damage and membrane damage; and e) NO, intense green color represents high level of nitric oxide in roots of *B. juncea*. (**2**) In situ visualisation of *B. juncea* leaves indicating the generation of (**a**) H_2_O_2_ exposed with DAB, brown color indicating the H_2_O_2_ formation, (**b**) O_2_^−^ tagged with NBT, blue color signifying the production of O_2_^−^. (**c**) Lipid peroxidation uncovered with Schiff’s reagent, pink color representing the lipid peroxidation. (**d**) Membrane integrity tagged with Evan’s blue, blue color indicating the loss of membrane integrity in *B. juncea* L. seedlings. (Note: Cn—without CP control, CP—chlorpyrifos, EBL—24-epibrassinolide, Ma—*Pseudomonas aeruginosa* and Mb—*Burkholderia gladioli*).

**Figure 3:**
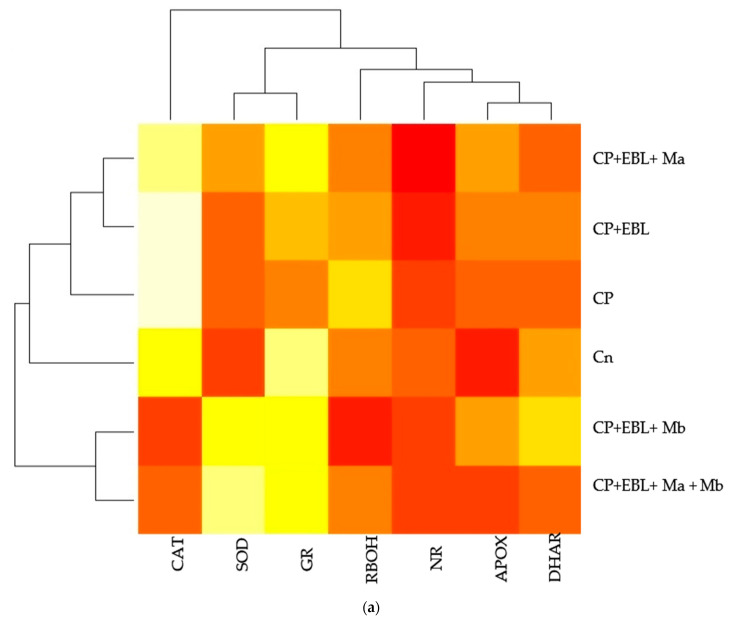

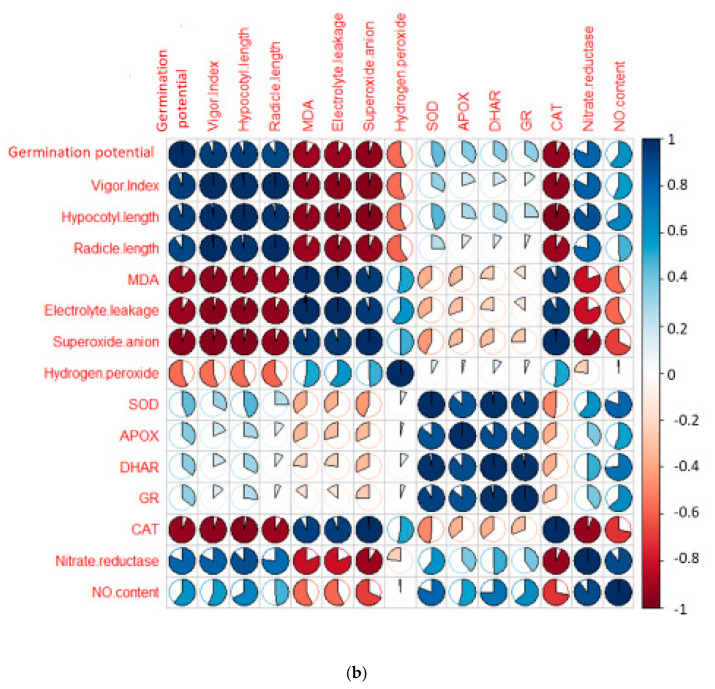

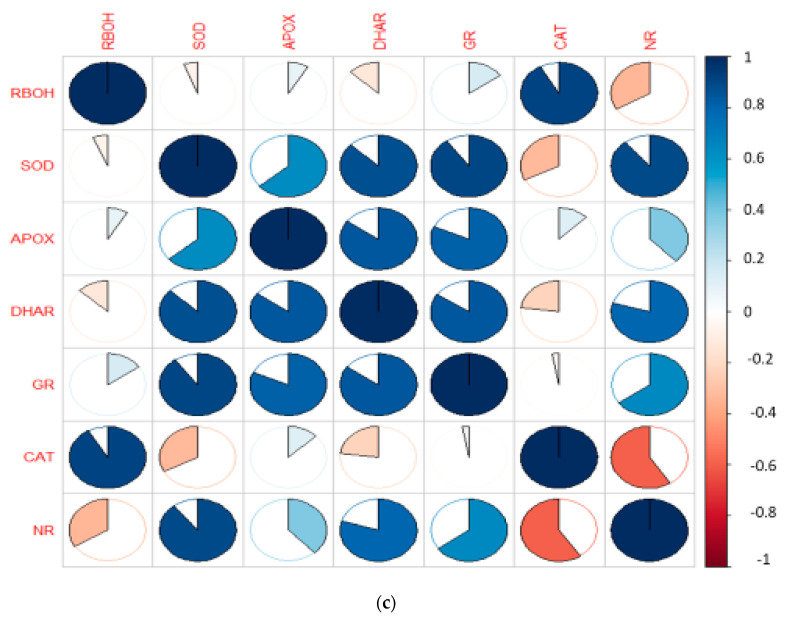
(**a**) Heatmap representing the effect of EBL and PGPRs on the gene expression of differentially expressed antioxidative defence enzymes and nitrate reductase on 10-day-old *B. juncea* L. seedlings grown under chlorpyrifos stress. The intensity of color expresses the intensity of transcript expressed. Seedlings without CP, EBL and PGPRs (Control, Cn), chlorpyrifos-treated (CP), chlorpyrifos with 24-epibrassinolide (CP + EBL), chlorpyrifos with 24-epibrassinolide and *Pseudomonas aeruginosa* (CP + EBL + Ma), chlorpyrifos with 24-epibrassinolide and *Burkholderia gladioli* (CP + EBL + Mb) and chlorpyrifos with 24-epibrassinolide and *Pseudomonas aeruginosa* and *Burkholderia gladioli* (CP + EBL + Ma + Mb). Pearson correlation analysis was executed to demonstrate the relationship of growth parameters with oxidative stress markers and defence enzymes at biochemical (**b**) and transcript level (**c**). Scale on the right end represents the color variation in which blue scale represents the positive and pink scale represents the negative correlation. (In-house script: http://www.sthda.com/english/wiki/visualize-correlation-matrix-using-correlogram. 2 December 2020 https://www.datanovia.com/en/lessons/heatmap-in-r-static-and-interactive-visualization) accessed on 2 December 2020.

CP-treated seedlings of *B. juncea* exhibited a large amount of oxidative stress due to anomalous production of O_2_^−^ and H_2_O_2_. CP toxicity showed a drastic rise in the level of O_2_^−^ by 70.5%, and H_2_O_2_ by 45.7% when comparing with and without CP seedlings. Treatment of EBL and PGPRs in CP-stressed seedlings decreases the activity of oxidative stress markers when compared with CP-treated only. However, a rigorous decline in their content (O_2_^−^ by 117.3%, Figure 1E, H_2_O_2_ by 69.71%, Figure 1F) was uncovered in seedlings raised with the combined effect of EBL and Mb. Supplementation of both the microbial strains (Ma, Mb) along with EBL also showed an acute decrease in these species when compared with CP-treated only. Moreover, with two-way ANOVA, a significant change was observed in the O_2_^−^ and H_2_O_2_ contents among different combinations of treatments. The fluorescent staining of roots of *B. juncea* seedlings with 2,7-dichloroflourcein diacetate (DCF-DA) indicates an intense green color in CP-stressed roots (Figure 2(1)a), which was in good agreement with the above uncovered H_2_O_2_ content. The annotations obtained from histochemical analysis for O_2_^−^ and H_2_O_2_ in leaves with NBT and 3,3-diamino benzidine (DAB) were also in conformity with the results obtained. Dark brown staining and deep blue coloration in Figure 2(2)a,b of *B. juncea* leaves under CP treatment was observed. Seed priming with EBL and inoculation of PGPRs alone, i.e., EBLMa and EBLMb and in combination (EBLMaMb), alleviated the stress by reducing the O_2_^−^ and H_2_O_2_ content as shown by sheen pattern in the Figure 2(2)a,b. The fluorescent staining of roots of *B. juncea* seedlings with 2,7-dichloroflourcein diacetate (DCF-DA) were also in good agreement with the above uncovered H_2_O_2_ content. Pearson correlation analysis (Figure 3b) also found that the growth of the seedlings showed a negative correlation with O_2_^−^ and H_2_O_2_. The results conclude that CP stress causes severe oxidative damage in *B. juncea* seedlings, which was mitigated when EBL and PGPRs were administered, highlighting the ameliorative role of EBL and PGPRs.

### 3.3. Effect of EBL and PGPRs on Lipid Peroxidation and Membrane Permeability in Brassica juncea Seedlings Treated with CP

The effect of CP on lipid peroxidation and membrane permeability on *B. juncea* seedlings was inspected by measuring the content of MDA and EL. CP-treated seedlings exhibited a sharp increase in the MDA and EL content when compared with seedlings without CP, i.e., control. However, when CP-treated seedlings were cultured with EBL, a 60.8 and 51.5% recovery in MDA and EL content (Figure 1G–H) was observed. As compared with the CP-stressed seedlings, MDA and EL showed maximum peaks by 124.98 and 98.8%, respectively, when treated with EBL and Mb (Figure 1G–H). A significant difference was recorded when the groups were compared with two-way ANOVA. The histochemical observation of the nuclear damage and membrane damage, with PI, DAPI and their interlay (Figure 2(1)b–d), showed the bright red and blue fluorescence in the case of CP-stressed seedlings. PI intercalates with the nucleic acids and forms a fluorescent complex which, in the case of living cells, is impermeable and forms an outline over them, while in damaged and dead cells, it passes and stains the nuclei. However, DAPI cannot easily pass through the membranes of the living cells, while in the case of CP stress, bright blue fluorescence appeared, indicating membrane damage. Lipid peroxidation and membrane permeability by Schiff’s reagent and Evan’s blue also coincided with the biochemical observations. Seedlings that had been treated with CP had a deep pink tint and a dark blue colouring (Figure 2(2)c,d), which on inoculation with PGPRs and EBL pre-treatment showed a shallow staining pattern. Pearson correlation analysis (Figure 3b) also showed a negative correlation between the overall growth of the seedlings and MDA and EL content, indicating a negative effect of MDA and EL on seedling growth. It is possible to conclude that CP stress leads to lipid peroxidation, which causes membrane leakage and alters the plant growth. EBL and PGPR therapy reduced the damage, implying that they play a positive role in reducing lipid peroxidation and membrane leakage.

### 3.4. Effect of EBL and PGPRs on Antioxidative Enzymes in Brassica juncea Seedlings Treated with CP

**Figure 4:**
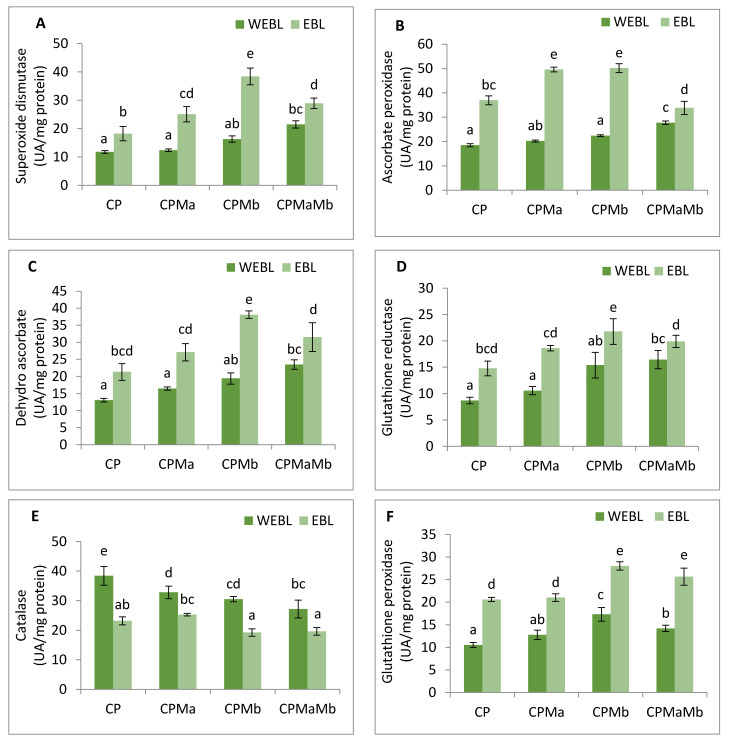
Effect of seed priming with 100 nM of 24-epibrassinolide (EBL) and plant-growth-promoting rhizobacteria (*Pseudomonas aeruginosa* (Ma, 10^9^ cells/mL) and *Burkholderia gladioli* (Mb, 10^9^ cells/mL)) on the activity of (**A**) SOD, (**B**) APOX, (**C**) DHAR, (**D**) GR, (**E**) CAT and (**F**) GPOX of 10-day-old *B. juncea* L. seedlings under chlorpyrifos (CP) toxicity. Data are presented as mean of three replicates ± s.d. (standard deviation). Means were compared using two-way analysis of variance followed by Tukey’s post-hoc test (*p* < 0.05). Different superscripts indicate significant difference between the treatments. (Note: WEBL = *without 24-epibrassinolide*, EBL = *24-epibrassinolide*).

In the current study, the antioxidative enzyme activities were analysed. SOD, APOX, DHAR, GR and GPOX showed increment in the activities, while CAT showed a decrease in response to CP treatment. In CP-treated seedlings, the activity of SOD was found to be enhanced by 1.41-fold, which upon seed priming with EBL along with supplementation of Ma and Mb alone and in combination, i.e., EBL Ma Mb, showed an increase in the activity by 2.127-fold, 3.25-fold and 2.451-fold (Figure 4A). The maximum peak in SOD activity was observed in EBL-primed seeds along with Mb supplementation. Treatment with EBL along with Ma and Mb alone and in combination significantly enhanced the APOX activity by 2.681-, 2.713- and 1.828-fold when compared with the CP-treated seedlings (Figure 4B). DHAR activity was increased by 2.52-fold in CP-treated seedlings over without-CP control seedlings which upon application of EBL along with Ma and Mb alone and in combination showed a significant increase by 2.072-fold, 2.917-fold and 2.413-fold. However, the maximum increment in the activity of the DHAR was observed in EBL along with Mb treatment (Figure 4C). Activity of GR was also analysed, and an increase of 2.062-fold was observed in response to CP treatment. Application of EBL and Mb showed a maximum increase by 2.505-fold when compared with the CP-treated seedlings (Figure 4D). The activity of CAT was observed to be increased in CP-treated seedlings by 2.149-fold when compared with the without-CP seedlings. However, a decrease in the activity was observed when treated with only EBL, EBL and Ma, EBL and Mb and a combination of EBL, Ma and Mb, by 0.605, 0.658, 0.5 and 0.51, respectively (Figure 4E). GPOX activity in CP-treated seedlings was observed and showed an increase by 1.413-fold over the control seedlings. The combined effect of EBL and Mb showed the maximum increase in the GPOX activity of 2.66-fold (Figure 4F). Pearson correlation (Figure 3b) analysis also showed that SOD, APOX, DHAR, GR and GPOX showed a positive correlation amongst themselves as well as with the seedling growth. The analysis of change in the enzyme’s activity using two-way ANOVA was also considered to be significantly different. These observations depict that EBL treatment along with inoculation of PGPRs in CP-stressed seedlings strengthens the antioxidative defence enzymes.

### 3.5. Effect of EBL and PGPRs on NO and NR Activity in Brassica juncea Seedlings Treated with CP

**Figure 5:**
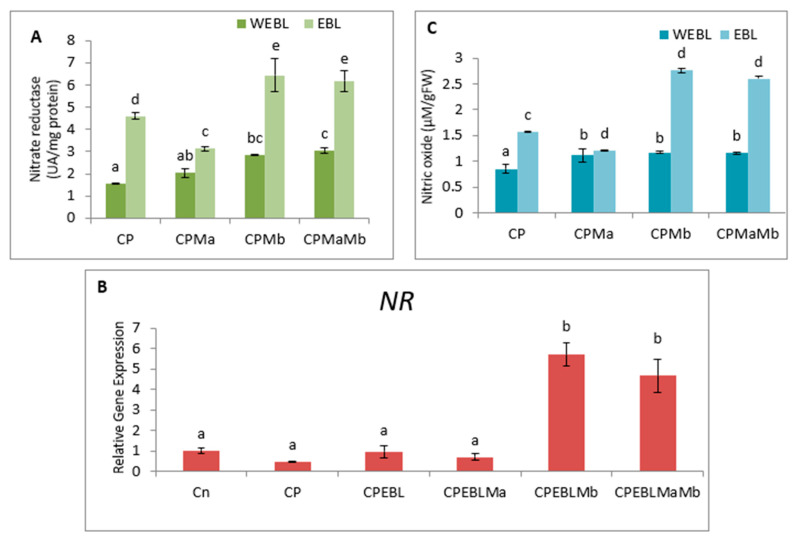
Effect of seed priming with 100 nM of 24-epibrassinolide (EBL) and plant-growth-promoting rhizobacteria (*Pseudomonas aeruginosa* (Ma, 10^9^ cells/mL) and *Burkholderia gladioli* (Mb, 10^9^ cells/mL)) on (**A**) Nitrate reductase activity and (**B**) its expression and (**C**) Nitric oxide (NO) level of 10-day-old *B. juncea* L. seedlings under chlorpyrifos (CP) toxicity. Data are presented as mean of three replicates ± s.d. (standard deviation). Means were compared using two-way analysis of variance followed by Tukey’s post-hoc test (*p* < 0.05). Different superscripts indicate a significant difference between the treatments. (Note: WEBL = *without 24-epibrassinolide*, EBL = *24-epibrassinolide*).

The observations on CP-exposed *B. juncea* seedlings show a decrease in NR enzyme activity (Figure 5A,B), as well as NO content (Figure 5C), by 0.297-fold and 0.623-fold over the without-CP control. In the present study, supplementation with PGPRs (Ma, Mb) and pre-treatment of EBL with PGPRs and in combination, i.e., EBLMaMb, enhanced the enzymatic activity of NR and NO. Treatment with EBL and Mb showed maximum enhancement of NR activity, and NO content was observed to be 4.21- and 2.76-fold greater as compared to CP alone (Figure 5A). The application of two-way ANOVA depicted a significant difference in the NR activity and NO content. The presence of NO content was also visualised by staining the roots of *B. juncea* seedlings with 4-amino-5-methyl-amino-2′, 7′ difluoroescein diacetate (DAF-FM DA). A shallow pattern of green color staining was observed in the CP-stressed roots, which upon supplementation with EBL and PGPRs showed dark fluorescence, which confirmed the NO presence in comparison to CP-treated alone (Figure 2(1)e). Pearson correlation analysis (Figure 3b) represented that NR and NO have positive correlation with each other as well as with *B. juncea* growth. The results indicate that the concurrent supplementation of EBL and PGPRs positively influences the NR activity, indicating the ameliorative role of EBL and PGPRs in CP stress.

### 3.6. Effect of EBL and PGPRs in Regulating the Expression of Antioxidative Defence-Related Genesin CP-Treated Brassica juncea Seedlings

**Figure 6:**
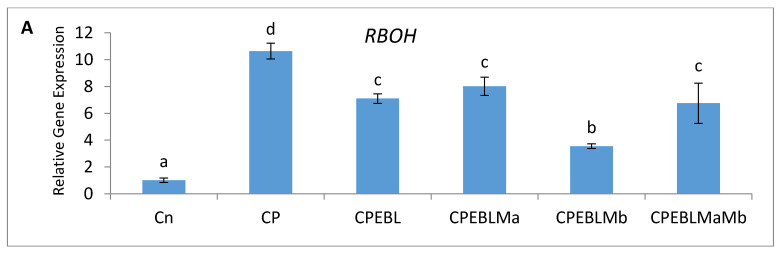

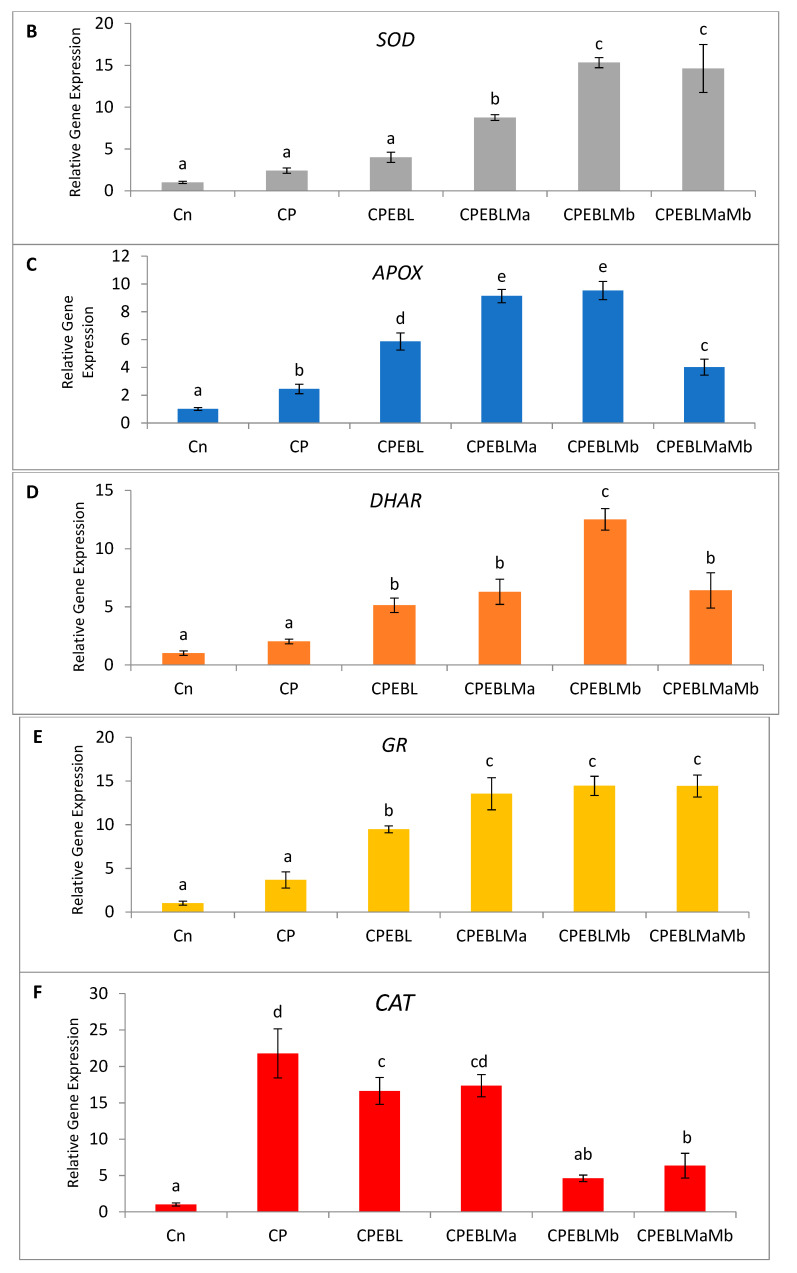
Effect of seed priming with 100 nM of 24-epibrassinolide (EBL) and plant-growth-promoting rhizobacteria (*Pseudomonas aeruginosa* (Ma, 10^9^ cells/mL) and *Burkholderia gladioli* (Mb, 10^9^ cells/mL)) on gene expression of (**A**) *RBOH, (***B**) *SOD, (***C**) *APOX, (***D**) *DHAR,* (**E**) *GR* and (**F**) *CAT* of 10-day-old *B. juncea* L. seedlings under chlorpyrifos (CP) toxicity. Data are presented as mean of three replicates ± s.d. (standard deviation). Means were compared using two-way analysis of variance followed by Tukey’s post-hoc test (*p* < 0.05). Different superscripts indicate significant difference between the treatments.

In this study, an increment in the expression of the RBOH1 (*respiratory burst oxidase1*) gene in CP-treated *B. juncea* seedlings was observed when compared with the control (without CP). The maximum decrease in the expression of RBOH1 of 3.55-fold was noted with EBL- and Mb-treated seedlings under CP stress in comparison to CP-treated only (Figure 6A). Additionally, in CP-treated seedlings, the antioxidative enzymes SOD (Figure 6B), APOX (Figure 6C), DHAR (Figure 6D), GR (Figure 6E) and CAT (Figure 6F) were analysed at the transcript level, which showed an increase in the expression of the aforesaid enzymes compared to control seedlings. Enhanced expression of SOD, APOX, DHAR and GR was observed in CP-treated seedlings when compared with EBL pre treated only, EBL pretreated along with PGPRs (Ma, Mb) alone i.e. EBLMa, EBLMb and in combination, i.e., EBLMaMb. Treatment of EBL along with Mb showed a greater maximum increase in SOD (6.33-fold), APOX (3.897-fold), DHAR (6.196-fold) and GR (3.937) (Figure 6B–F) than CP-stressed seedlings. However, a decreased expression of CAT (0.211-fold) was observed but maximum reduction was recorded with EBL- and Mb-treated seedlings relative to CP treatment only. In comparison with without-CP, i.e., control, seedlings, reduced NR expression was observed in CP-treated seedlings. Treatment of EBL along with Mb showed a maximum increase of 11.827-fold (Figure 6B) in NR activity when compared with CP-treated only. A heatmap shows that the combined effect of EBL along with PGPRs showed a maximum change in the defence system at the transcript level (Figure 3a). In Figure 3a, CPEBLMb and CPEBLMaMb clustered together in a family, as GR, NR and CAT in both the treatments showed almost similar variations. However, CPEBLMa and CPEBL clustered together in a group, as RBOH, NR, DHAR and APOX showed almost similar variations. Pearson correlation (Figure 3c) also showed a positive correlation among the expression of SOD, APOX, DHAR, GR and NR genes. However, CAT showed a negative correlation. A significant difference for RBOH, SOD, APOX, DHAR, GR, CAT and NR genes was also observed using one-way ANOVA. It was noticed through Pearson correlation analysis (Figure 3b–c) that in CP-stressed seedlings, overall growth of the plants was negatively correlated with germination potential, hypocotyl length and radicle length. Similarly, seedling growth was also negatively correlated with O_2_^−^, H_2_O_2_, MDA and EL while positively correlated with SOD, APOX, DHAR and GR. This correlation depicts a close relationship between growth of stressed seedlings, oxidative stress indicators and the enzymatic defence mechanism at the transcript level. These observations show that seed priming with EBL along with supplementation of PGPRs could be a useful approach in strengthening the enzymatic defence enzymes at the transcript level in CP-stressed *B. juncea* seedlings.

## 4. Discussion

Pesticide toxicity hampers the growth and development of the plant and monitoring these parameters keeps an eye on plant health, which has an unwavering impact on agricultural produce. In this investigation, under CP stress, the germination efficiency and ultimate development of *B. juncea* seedlings were observed to be significantly affected. The results obtained are in corroboration with those of Sharma et al. [80] on imidacloprid toxicity in *B. juncea* seedlings. Pesticide poisoning has been shown by several researchers to impair germination potential and change development patterns in *O. sativa* [12], *B. juncea* [81] and *Trigonella* [17]. However, the abatement in the growth of the seedlings under CP stress could be related to oxidative stress generated by increased reactive oxygen species (Figure 1E,F). The use of EBL in conjunction with Mb provided the best outcomes on germination potential, growth and overall development of the CP-stressed seedlings followed by co-inoculation of Ma and Mb with EBL. The importance of BRs in stimulating germination and growth has been investigated in *Triticum aestivum* [82], *O. sativa* [12], *B. juncea* [80,81,83,84], *Solanum lycopersicum* [85], *Arabidopsis thaliana* [86] and *Zea mays* [87]. In fact, it is now entrenched that BRs play an effective role in growth and development of the cell by stimulating H^+^-ATPases that are involved in the activation of enzymes required for loosening of cell walls [88]. BRs have the ability to regulate the expression of a large number of genes that control various metabolic pathways [89]. They also modulate the biosynthetic pathway of cellulose, sucrose synthase, xyloglucanendotransglucosylase/hydrolase, cell division and its expansion by regulating the key enzymes of growth at the transcript level [26,90]. They are also known to work in tandem with auxins to strengthen the cell elongation process [91]. 

Concomitantly, Jaiswal et al. [92] revealed that inoculating insecticide-stressed plants with plant-growth-promoting bacteria improved the seed germination. *Bacillus amyloliquefaciens*, a rhizobacterium, stimulated plant growth in *Arabidopsis thaliana* by growing lateral roots and root hair formation [93]. Supplementation of *Achromobacter xylosoxidans* and *Ochrobactrum* sp. in CP-stressed *Vigna unguiculata* significantly boosted the growth of the plant [94]. In fact, plant development can be aided by PGPRs even when the plant is stressed, by producing siderophores, growth-stimulating hormones, nitrogen fixation and phosphate solubilisation [95,96]. Plant–microbe interaction has shown that inoculation of PGPRs induces changes in hormone-mediated key plant genes [97,98]. Moreover, plant hormones also influence how plant–microbe interactions evolve [99]. A constructive role of BRs in arbuscular mycorrhizal (AM) association and nodulation was presented by McGuiness et al. [100,101,102]. In tomato, pea and rice, mutations in the BR synthesising cascade inhibited AM symbiosis [103], whereas foliar application of BRs in wheat encouraged AM colonisation [104]. The present study substantiates these observations, showing that BRs play a positive role in plant–microbe interactions. 

To address the possibility of oxidative stress-induced growth inhibition in *B. juncea*, we analysed the ROS content qualitatively and quantitatively in terms of H_2_O_2_ and O_2_^−^ in 10-day-old *B. juncea* seedlings grown in the presence of CP.The content of H_2_O_2_ and O_2_^−^ were observed to be enhanced with the CP treatment when compared with control only, whereas histochemical observations (Figure 2(1)a,b) are also in sync with biochemical evidence. A significant change in O_2_^−^ and H_2_O_2_ was also observed by Sharma et al. [105] in *O. sativa* under pesticide stress. In plants, RBOH1 (*respiratory burst oxidase homologue 1*) has been considered as the gene responsible for the production of reactive oxygen species under stress conditions [106,107,108] which showed significant upregulation in CP-stressed *B. juncea* seedlings when compared with the control in the present study. Oxidative stress is caused by an accumulation of activated oxygen molecules, which is caused by an uneven creation and detoxification cycle of reactive oxygen species (ROS) and is also synthesised as a stress signal [109]. In the current analysis, the greatest diminution in the O_2_^−^ and H_2_O_2_ content is observed in the seedlings when treated with EBL and Mb, followed by the combination of EBL and Ma and Mb. The damaging effect of oxidative burst caused by lipid membrane peroxidation and cellular destabilisation is then stimulated by the production of ROS. Our results unveil that CP-stressed seedlings also showed MDA accumulation (Figure 1G) which was further confirmed by histochemical studies reflecting the severity of damage caused in *B. juncea* by CP stress (Figure 2(2)c). The extent of nuclear and membrane damage caused due to CP toxicity in *B. juncea* roots was also validated using fluorescence microscopy (Figure 2(1)b–d). A similar increment in MDA content was observed upon treatment of CP in *O. sativa* [105], thiram in *S. lycoperscion* [110], tricyclazole and plethora in *Trigonellafoenum-graecum* [17] and imidacloprid and dichlorvos in cucumber [111]. Enhanced lipid peroxidation also exacerbates the level of electrolyte leakage in CP-stressed *B. juncea* seedlings and disrupts membrane integrity (Figure 1H and Figure 2(2)d). Our results reveal that the maximum decrease in O_2_^−^, H_2_O_2_, MDA and EL is observed in EBL- and Mb-treated *B. juncea* plants grown under CP treatment, that subsequently indicates the ameliorative role of EBL and Mb towards membrane damage. The results obtained are in abidance with Wang et al. [112], who accounted for the decrease in MDA upon EBL application in grapevine under chlorothalonil stress. Reduced ROS and MDA content were documented upon application of BR mimetics by Liu et al. [113] in maize plants under nicosulfuron toxicity. Concomitantly, applications of *Bacillus subtilis* in *Solanum tuberosum* under stress conditions increase the membrane stability and decrease the O_2_^−^, H_2_O_2_ and MDA content [114] by strengthening the antioxidative defence enzymes. Plants are relentlessly integrating these signals to calibrate resourcefully the safety program under stressed conditions. They have a well-developed first line of defence, which involves an antioxidative defence mechanism based on enzymes. CP-induced ROS burst significantly increased the activity of SOD, APOX, DHAR, GR, CAT and GPOX. In CP-treated *B. juncea* seedlings, application of EBL and Ma and Mb, alone and in combination with EBL, increased the activities of all enzymes except CAT. The increased activities of the defence enzymes could be responsible for the decrease in ROS accumulation after treatment with EBL and PGPRs. SOD belongs to a key class of antioxidant proteins, which serves as a first line of defence against a variety of stresses. SOD carries out the catalytical dismutation of O_2_^−^ to molecular oxygen and H_2_O_2_ [115]. In the current study, application of EBL and Mb in combination showed the maximum decrease in O_2_^−^ content, which might be due to the increased SOD activity at the transcript level. However, scavenging of accumulated H_2_O_2_ depends on the balance of APOX, CAT and GPOX. Seed treated with EBL and Mb showed the maximum increase in the expression of APOX activity, which could be the possible reason, as the first step in the scavenging cycle of H_2_O_2_ includes reduction of H_2_O_2_ in the presence of APOX. However, enhanced GPOX activity also been observed in EBL- and Mb-treated CP-stressed *B. juncea* seedlings. A negative correlation between the ROS and SOD, APOX, GR and DHAR was observed (Figure 3c). However, decreased activity of CAT was observed upon treatment with EBL and Ma and Mb alone and in combination. A positive correlation between RBOH and CAT was found and this may be attributed to the altered defence system. Similar findings were documented by Hakeem et al. [116] in *Fagopyrum kashmirianum*. The decrease in the activity of CAT could be due to excess ROS production, interference with the sub-units or altered synthesis [117] or change in enzymes balance promptly triggering the compensatory mechanisms (i.e., APOX and GPOX) [118]. An investigation carried out by Cuypers et al. [119] extended our knowledge regarding APOX involvement in H_2_O_2_ detoxification. In CP-treated seedlings, the maximum increase in DHAR and GR was observed at the mRNA level upon treatment with EBL and Mb. A similar rise in BR’s dependent enzymatic defence activity was observed in tomato [120], rice [105], grapevine [112] and mustard [80,81]. A stimulatory effect of PGPRs was also recorded in *Glycine max* [121], rice [122] and tomato [48]. Increased expression of Asada–Halliwell pathway genes with EBL and PGPRs decreasing the membrane peroxidation by scavenging ROS, allowing the plant cell to maintain structural and functional stability under CP stress. 

In the present investigation, CP-stressed *B. juncea* seedlings showed a decrease in NR activity (Figure 5A) and NO content (Figure 5C). This study coincides with Gupta and Seth [22], who also documented the decrease in NR activity in *B. juncea*, which lead to increased MDA and H_2_O_2_ production. It might be possible that increased production of MDA and H_2_O_2_ in CP-stressed seedlings could be due to NR activity also. Application of EBL along with Ma and Mb alone and in combination upregulates the expression of the NR gene (Figure 5B), resulting in subsequent enhancement in NO level. Fluorescent tagging of *B. juncea* roots (Figure 2(1)e) for NO also showed an increase upon treatment with EBL and PGPRs. Further, upon addition of EBL and Mb, the most significant rises in NR and NO activity were observed. In wheat seedlings, the ameliorative role of NO in regulating the oxidative stress was well documented by Tripathi et al. [123]. BRs have the ability to induce NO synthesis in a ROS-dependent pathway, which accumulates in plant cells and acts as a signal for the activation of the stress tolerance response [124]. This backs up the idea that higher NO levels help to balance the plant’s defensive mechanisms during times of stress. The results are supported by the Pearson correlation analysis which showed a positive correlation between NR and SOD, APOX, DHAR and GR. During the interaction between plant and microbes, production of NO was observed [125,126]. As a result, the current study investigates the relationship between NO and the CP stress-relieving effects of EBL and PGPRs. Graphical representation of gene expression by heatmap placed the combination of EBL and Mb, as well as EBL and Ma and Mb treatment in the same group, representing the maximum ameliorative potential of these treatments in comparison to others (Figure 3a). BR- and PGPR-mediated enhancement in the antioxidative defence mechanism might be due to their regulation of the genes at the transcriptional/translational level, that modulates the activation or de novo synthesis [83]. As a result, our findings suggest that plant hormone-dependent plant–microbe interaction may help CP-stressed seedlings by strengthening their defence mechanisms at the transcriptional stage. 

## 5. Conclusions

The results from the present study show that CP toxicity reduced the germination potential and overall growth of *B. juncea* seedlings due to an oxidative burst caused by increased reactive oxygen species. Oxidative stress also reduces the production of NO which acts as a signal molecule, disintegrates the membrane integrity and causes cell injury. However, seed priming with 24-epibrassinolide, and supplementation of *P. aeruginosa* and *B. gladioli* stimulates the plant defence by strengthening the ROS-scavenging antioxidative defence enzymes. The findings also shed light on the role of NO production mediated by nitrate reductase in plant signalling to trigger antioxidative defence enzymes. With current knowledge, the effectiveness of plant hormone-mediated interactions between plants and microbes in reducing pesticide toxicity in plants has increased. The results are critical from an agronomic standpoint for managing pesticide toxicity in plants and increasing produce growth. 

## Figures and Tables

**Table 1:** Primer sequences of various genes for qRT-PCR in the present study.

**Gene and Gene ID**	**Sequences**
*Actin* (KM881,428.1)	Forward Primer 5′ ACTGGTATTGTGCTTGACTCTG3′ Reverse Primer 5′ AGCTTCTCTTTAATGTCACGGAC3′
*SOD* (AF540,558.1)	Forward Primer 5′ CACATTTCAACCCTGATGGTAA3′ Reverse Primer 5′ ACAGCCCTTCCGACAATA3′
*APOX* (AF038,839.1)	Forward Primer 5′ CCACTTGAGACAGGTGTTACTA3′ Reverse Primer 5′ TCCTTGAAGTAAGAGTTGTCGAAA3′
*DHAR* (AF536,330.1)	Forward Primer 5′ CTGGATGAGCTTAGTACATTCAAC3′ Reverse Primer 5′ GGAAAGAAAGTGAATCTGGAACA3′
*GR* (AF349,449.1)	Forward Primer 5′ GATGCAGCGCTTGATTTAC3′ Reverse Primer 5′ TCCCTAACGTCTTCATCAAACC3′
*CAT* (AF104,451.1)	Forward Primer 5′ GTTCGACTTTGACCCACT3′ Reverse Primer 5′ ATCCCAGGAACAATGATAGC3′
*NR* (XM_022711045.1)	Forward Primer 5′ GGTGGAGGTGACTCTAGATG3′ Reverse Primer 5′ TCGAACCGCAACGTCTTTA3′

## Data Availability

No supplementary data available.
